# Artificial vision models for the identification of Mediterranean flora: An analysis in four ecosystems

**DOI:** 10.1371/journal.pone.0327969

**Published:** 2025-09-05

**Authors:** Parminder Kaur, Anna Grassi, Federica Bonini, Barbara Valle, Marina Serena Borgatti, Giovanni Rivieccio, Agnese Denaro, Leopoldo de Simone, Emanuele Fanfarillo, Paolo Remagnino

**Affiliations:** 1 Department of Computer Science, Durham University, Durham, United Kingdom; 2 Department of Agricultural, Food and Environmental Sciences, University of Perugia, Perugia, Italy; 3 NBFC, National Biodiversity Future Center, Palermo, Italy; 4 Department of Life Sciences, Università Degli Studi di Siena, Siena, Italy; 5 Department of Biosciences, Università Degli Studi di Milano, Milano, Italy; 6 Department of Chemical, Physical, Mathematical and Natural Sciences, Università di Sassari, Sassari, Italy; 7 Department of Agricultural Sciences, University of Sassari, Sassari, Italy; Al Baha University, SAUDI ARABIA

## Abstract

Object identification has been widely used in several applications, utilising the annotated data with bounding boxes to specify each object’s exact location and category in images and videos. However, relatively little research has been conducted on identifying plant species in their natural environments. Natural habitats play a crucial role in preserving biodiversity, ecological balance, and overall ecosystem health. So, effective monitoring of habitats is necessary for safeguarding them, and one way of doing this is by identifying the typical and early warning plant species. Our study quantitatively evaluates the performance of six popular object detection models on our dataset collected in the wild, comprising various plant species from four habitats: screes, dunes, grasslands, and forests. The dataset employed in this work includes the data collected by human operators and the quadrupedal robot ANYmal C. The pre-trained object detection models have been chosen for experiments, and they are fine-tuned on our dataset to achieve better performance. These models incorporate two one-stage (RetinaNet and YOLOv8n), two two-stage (Faster RCNN and Cascade RCNN), and two transformer-based detectors (DETR and Deformable DETR). Extensive experimentation has been performed on the four habitat datasets by applying class balancing and hyperparameter tuning, and the obtained results are discussed.

## Introduction

Habitats are essential in preserving biodiversity, reflecting the intricate interplay between living organisms and their surrounding environment. With the growing impact of human activities (deforestation, fossil fuel burning, pollution, trampling, etc.) on natural habitats, it has become essential to have precise and timely information regarding the condition of ecosystems. Recognised as pivotal by various researchers [1–5], habitats have been singled out by the European Commission (EC) as essential targets for efficiently assessing the state of nature conservation, as outlined in the 92/43/EEC “Habitats" Directive [6,7]. This directive stands as a cornerstone of European biodiversity conservation policy [8,9], notably through its Annex I, which identifies “Natural habitat types of Community interest whose conservation requires the designation of Special Areas of Conservation" [10]. In Europe, the conservation status of Annex I Habitats is scheduled for monitoring every six years, guided by official reference guidelines established at both European and national levels [11–13]. These guidelines encompass criteria such as the habitat’s distribution, structure, functions, and the conservation status of its ’typical’ species, which are species serving as indicators for habitat conservation status [6]. These ’typical’ species act as proxies, offering insights into environmental changes, including those known as ‘sentinels’ [14–16].

**Fig 1:**
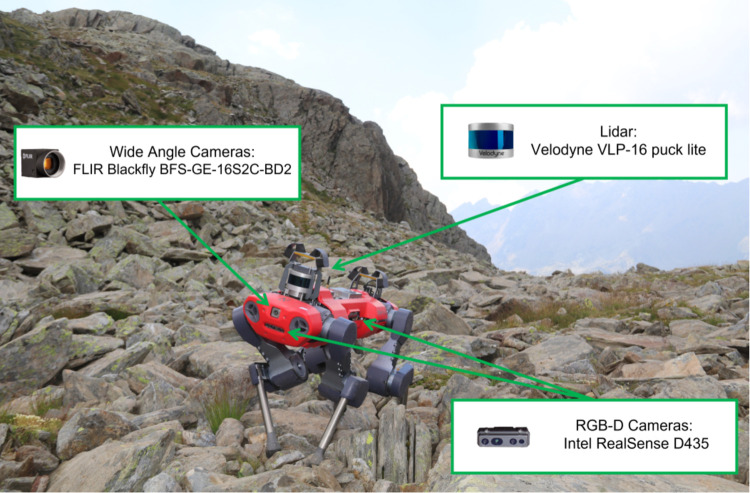
The quadruped robot ANYmal C deployed in the screes habitat [19]. ANYmal C is equipped with various sensors, including four Intel RealSense D435 RGB-D cameras for capturing high-resolution images. These sensors enable a large-scale and time-effective data acquisition that can be later used for habitat monitoring.

While artificial intelligence is increasingly employed across various applications, monitoring natural habitats predominantly relies on field observations by human experts, particularly in terrestrial environments [17]. However, several challenges hinder the effective monitoring of habitat conservation status as outlined in the Habitats Directive (92/43/EEC) [6,7]: (1) reliance on human operators as the primary monitoring option, (2) subjectivity introduced by human involvement may lead to inconsistency, and (3) limited time windows for habitat monitoring, requiring additional professional surveyors with the increase in habitat numbers. To address these challenges, leveraging state-of-the-art deep learning techniques can automate habitat monitoring processes or support human experts in the field. So, we curated our image data of target species, including typical species (TS), alien species (AS), and early warning species (EWS), from four habitats: screes, dunes, grasslands, and forests. The presence of TS signifies a favourable habitat conservation status, whereas the presence of EWS and AS indicates potential threats to habitat health [17]. This dataset used in the current experimental analysis is a combined data collected by both human operators and the quadrupedal robot ANYmal C [18]. Plant experts and robotics engineers collected data using the quadrupedal robot ANYmal C during habitat monitoring missions. Fig 1 shows the quadrupedal robot ANYmal C in the screes habitat and highlights the sensors utilised for data acquisition for multiple tasks [19]. Out of all the sensors, the RGB-D cameras are specifically used for acquiring data used in this study.

After data collection, domain experts annotated all the human and robot-collected images using bounding box annotations with the help of *Labelbox* tool [20] to prepare it for automatic plant species localisation and classification. Our motive is to monitor the natural habitat conservation status by identifying the target species within four habitats to aid humans in assessing the habitat conservation status. So, this article presents a comprehensive quantitative analysis of six object recognition models on our plant species dataset from four habitats, namely, screes, dunes, grasslands, and forests, to visualise the models’ (pre-trained on COCO dataset [21]) performance. The six models include two one-stage object detectors: *YOLOv8* [22] and *RetinaNet* [23]; two two-stage detectors: *Faster RCNN* [24] and *Cascade RCNN* [25]; and two transformer-based detectors: *DETR* [26]and *Deformable DETR* [27]. These deep learning models are chosen for their unique strengths, for instance, one-stage detectors for their speed and efficiency, two-stage detectors for their high accuracy and refined object localisation, and transformer-based models for their ability to handle complex scenes and robust feature extraction. This enabled us to address the diverse challenges in plant species identification, such as class imbalance, overlapping objects, and the need for precise localisation to some extent. YOLOv8 has been implemented separately from other detectors, which are implemented in the *MMDetection 2.x* toolbox [28]. The data annotations were exported using a Python script from the Labelbox tool in the COCO format. These annotations are converted to the YOLO format for YOLOv8 model execution. This study mainly focuses on the performance analysis of the six detection models on our plant species dataset collected in the wild by human operators and quadrupedal robot ANYmal C.

### Contributions

The significant contributions of the proposed study are as follows:

1. An extensive experimental analysis of six object detectors (belonging to three different types) for identifying plant species in four diverse habitats.
2. Performance evaluation of the object detection methods, excluding YOLOv8, after introducing class balancing using *ClassBalancedDataset* wrapper in MMDetection toolbox [28].
3. YOLOv8 efficacy analysis on test data from all four habitats after performing hyperparameter tuning. The implementation details of our experimental study are available at https://github.com/parminder1050/Plant_species_identification.git link.

## Related work

This section includes various research works in diverse application areas, such as plant disease detection, identification of active landslides and marine ship targets, identification of plant and weed species, etc., incorporating the six methods (Faster RCNN, Cascade RCNN, RetinaNet, YOLOv8, DETR, and Deformable DETR) used in this experimental study. Table 1 presents a broad qualitative comparison of the utilised object detection methods based on specific parameters.

**Table 1:** Qualitative comparison of object detection methods used in the study based on various parameters.

Methods/ Parameters	Faster RCNN	Cascade RCNN	RetinaNet	YOLOv8	DETR	Deformable DETR
Architecture	Two-stage	Two-stage	One-stage	One-stage	Transformer-based	Transformer-based
Region Proposals	RPN	Cascade of RPNs	None	None	None	None
Speed	Medium	Medium	Fast	Very Fast	Medium	Medium
Accuracy	Medium-High	High	Medium-High	High	Medium-High	Medium-High
Flexibility	Moderate	Moderate	Limited	Limited	Limited	Limited
Training Time	Moderate	Long	Short	Moderate	Long	Long
Inference Time	Moderate	Moderate	Fast	Very Fast	Moderate	Moderate
Memory Usage	High	High	Moderate	Moderate	High	High

Detecting plant diseases is a challenging task due to: (1) similar appearance of normal and diseased plant leaves; and (2) significant variations in colour, intensity, shape, and size across both the background and foreground of plant images. To overcome these issues, Nawaz et al. [29] introduced Faster RCNN employing a VGG-19 network to detect and categorise different plant diseases. The proposed technique was tested on the PlantVillage data repository and found promising in recognising plant disease under diverse image-capturing scenarios. Real-time Faster RCNN has been utilised in [30] for tomato plant leaf disease detection. Saleem et al. [31] extended their previous research of weed detection by performing an in-depth analysis of the Faster RCNN technique with ResNet-101 as it was the best performing method among others. After conducting a deep investigation, they developed a Faster RCNN enhancement by incorporating diverse anchor box scales and aspect ratios to improve the weed detection performance, especially for *Chinee* apple weed class. Identification of diabetic retinopathy has been performed using Faster RCNN with feature fusion (a combination of multiple image feature extractors) [32]. Cia at. al. [33] employed an improved Faster RCNN based on attended ResNet-34 and feature pyramid networks for identifying active landslides over vast areas.

Cascade RCNN has been used for identifying the marine ship targets [34]. It is challenging to detect the ship targets due to harsh environments such as fog, snow, and rain, and also, the targets are usually blocked or small in size. Cascade RCNN is compared with Faster RCNN and has been found to perform better. Mo et al. [35] proposed an optimisation model for defect detection on steel surfaces, utilising Cascade RCNN, termed CR-SSDD. This model is designed to effectively identify various categories of defects across multiple scales on the steel surface. Cao et al. [36] proposed an improved Cascade RCNN method for detecting different ceramic tile surface defects in diverse texture backgrounds. Improved Resnet and feature pyramid network versions are utilised to identify low-contrast and small-scale ceramic tile defects. Tang et al. [37] introduced a Cascade RCNN with ResNet-50 backbone based approach to identify the athletes in the soccer videos, which is a burdensome task due to blurring and occlusion issues. A hybrid attention module has been proposed to enhance the occluded and blurred features in the feature pyramid. Cao et al. [38] performed a multi-scale face mask identification using an improved Cascade RCNN technique dubbed MFMDet. It utilises a recursive feature pyramid to handle multi-scale features extracted by the backbone network. This approach enhances the receptive field and improves the representation of multi-scale features, thereby achieving effective detection, particularly for small objects.

Weeds pose a significant threat to both the yield and quality of crops. Automating the weed detection process is challenging due to the overlapping presence of crops and weeds. So, Peng et al. [39] proposed a technique based on RetinaNet to detect the weeds in the paddy field. A dataset consisting of images of rice plants and various weed species was created through on-site photography and web crawling. It encompasses rice plants along with eight different categories of weeds. Vecvanags et al. [40] proposed two algorithms based on RetinaNet and Faster RCNN to detect the ungulates in the images. A new dataset of wild ungulates has been composed from Latvia. Training optimisation and change in algorithm performance with data augmentation have also been discussed. Miao et al. [41] proposed and improved lightweight RetinaNet for fast and accurate ship detection in Synthetic Aperture Radar (SAR) images. The initial aspect ratios of the architecture have been adjusted using the K-means clustering method. Mahum et al. [42] introduced a Lung-RetinaNet technique based on RetinaNet to detect lung tumours.

Accurate identification of plant traits is essential for timely monitoring and assessing their growth and readiness for harvest. Solimani et al. [43] proposed the use of the YOLOv8 model to proficiently detect flowers, fruits, and nodes in tomato plants. They have also addressed the challenge of uneven distribution of samples, potentially resulting in misclassification and disruptions in model recognition. Brucal et al. [44] performed a tomato leaf disease detection using YOLOv8 to identify nine diseases. Yang et al. [45] utilised YOLOv8 for plant leaf detection, reducing the background interference for further leaf segmentation tasks. Wang et al. [46] performed an automatic vegetable disease detection in a greenhouse environment using an improved YOLOv8 model. The proposed approach shows promising results on the self-composed vegetable disease detection dataset. Uddin et al. [47] proposed a Cauli-Det approach based on an improved YOLOv8 model for automatic cauliflower disease localisation and classification. They utilised images taken with smartphones and handheld devices for experimentation. A fine-tuned pre-trained YOLOv8 architecture has been used to detect regions affected by diseases and extract spatial features for localising and categorising the diseases.

Zhong et al. [48] proposed a DETR-based human-object interaction detection technique. DETR’s robustness has been increased by identifying hard-positive queries, which are required to make accurate predictions using partial visual cues. Kumar et al. [49] have utilised the DETR model for target detection. The DETR was fine-tuned on the custom dataset and showed a noticeable improvement in terms of the number of training epochs, both visually and statistically. Yuan et al. [50] performed sea cucumber detection using YOLOv5 and DETR and compared the performances of both architectures. The CNN-based method is unreliable in accurately identifying vertebral levels. So, Tang et al. [51] proposed the use of DETR for lamina detection, spine curve measurement, and vertebral level identification. The execution involved three significant goals: (1) automatic identification of lamina pairs, (2) Spinal curvature assessment, and (3) vertebral level recognition. Cheng et al. [52] have incorporated the use of DETR into insulator defect detection by automatically detecting faults in UAV-captured insulator images. Transfer learning techniques were employed to train the high-performing DETR model with a minimally collected insulator image dataset. An improved loss function was also integrated into the model to compensate for DETR’s limitations in detecting small objects at precise scales.

Wang et al. [53] utilised a deformable DETR for farmland obstacle detection from the UAVs’ perspective. Deformable DETR somewhat lacks the capability to capture long-range dependencies constrained by local receptive fields and local self-attention mechanisms. So, global modelling capability (inspired by non-local neural networks) has been incorporated into the front end of ResNet to improve the Deformable DETR performance. Airport security systems typically use radar-based detection technology to monitor UAVs and birds within the clearance area. However, they often struggle to accurately identify the type and number of UAVs as well as the size and quantity of birds. In light of this issue, Shanliang et al. [54] proposed a Deformable DETR based detection method for UAVs and birds to enhance airport supervision of the clearance area and mitigate safety risks. Wang et al. [55] proposed a students’ classroom behaviour detection system, which is based on Deformable DETR with Swin Transformer and light-weight Feature Pyramid Network. Utilising a feature pyramid structure enabled the system to efficiently handle multi-scale feature maps extracted by the Swin Transformer, thus enhancing detection accuracy for targets of varying sizes and scales.

## Methodology

This section comprises various subsections, such as Techniques, Model training, Evaluation metrics, Dataset, and Experimental setup, related to our experimental study. The *Techniques* subsection incorporates a detailed description of the three broad model categories (Two-stage detection, One-stage detection, and Transformer-based detection algorithms) and the six methods under these categories (two of each type). The other subsections provide the implementation details and the dataset description.

### Techniques

1. **Two-stage detectors**: Two-stage detection algorithms are a class of object detection algorithms characterised by two primary stages: region proposal and object detection. In the initial stage, these algorithms generate a collection of candidate object regions or proposals within the input image. Subsequently, they conduct classification and refinement of these proposals to identify objects and their precise locations. These algorithms typically amalgamate various techniques, encompassing feature extraction, region proposal, classification, and bounding box regression. The two two-stage detectors used in this study are as follows:
  (a) *Faster RCNN*: Faster RCNN (Region-based Convolutional Neural Network) [24] revolutionised object detection by integrating Region Proposal Networks (RPNs) directly into the detection pipeline. This two-stage approach generates region proposals and fine-tunes them through a shared convolutional backbone, enabling streamlined end-to-end training. By leveraging anchor boxes for proposal generation and parameter sharing, Faster RCNN achieves notable enhancements in both accuracy and speed, facilitating real-time object detection across diverse applications.
  (b) *Cascade RCNN*: Cascade RCNN [25] elevates object detection capabilities by utilising a cascade of detectors, which iteratively refine object proposals. This multi-stage approach involves progressively filtering out false positives and improving localisation accuracy. Cascade RCNN comprises multiple stages, each featuring a dedicated detector trained to address specific aspects of the detection task. During inference, proposals meeting the threshold in the initial stage undergo further refinement and evaluation in subsequent stages. Through the iterative refinement of object proposals, Cascade RCNN mostly achieves superior detection accuracy compared to single-stage detectors.
2. **One-stage detectors**: One-stage detection algorithms predict bounding boxes and class labels for objects within an input image in a single network pass. In contrast to two-stage detection algorithms, they do not require a separate region proposal step. Typically comprising a single neural network architecture, these algorithms take an input image and produce a collection of bounding boxes, along with their associated class labels and confidence scores. They optimise an end-to-end objective function, which integrates classification and localisation losses, enabling them to directly predict object locations and categories from the input image. The two one-stage detectors utilised in the current study are as follows:
  (a) *RetinaNet*: RetinaNet [23] introduces a single-stage object detection framework to overcome challenges encountered in object detection tasks, including class imbalance and localisation accuracy. Key to RetinaNet’s success is its novel focal loss function, which effectively handles the imbalance between foreground and background samples during training. The network architecture encompasses a feature pyramid network (FPN) backbone for multi-scale feature extraction, coupled with convolutional layers that predict class probabilities and bounding box offsets at each feature pyramid level.
  (b) *YOLOv8*: Introduced by the Ultralytics team [22], YOLOv8 represents the latest iteration of the renowned YOLO series of detection algorithms. It is an open-source, cutting-edge model distributed under the General Public License [56]. Following minor modifications to the YOLOv3 model, Glenn Jocher introduced YOLOv5 [57], paving the way for YOLOv8, an enhanced iteration of YOLOv5. YOLOv8 introduces numerous improvements to enhance detection accuracy, speed, and robustness. It adopts a deep neural network architecture capable of predicting bounding boxes and class probabilities directly from full-sized input images in a single pass. Notable enhancements in YOLOv8 include anchor-free detection, mosaic augmentation, and updates to the convolution blocks within the model, such as replacing the C3 module with the C2f module. YOLOv8 model structure summary and the enhancements from YOLOv5 can be found in [58].
3. **Transformer-based detectors**: Transformer-based detection algorithms employ transformer architectures as the foundation for feature extraction and processing. Originally devised for natural language processing tasks, transformers have recently gained popularity in computer vision applications, particularly object detection. These algorithms harness the transformer architecture, comprising self-attention mechanisms and multi-layer perceptrons, to effectively capture spatial relationships and dependencies among image features. Unlike conventional convolutional neural networks (CNNs), transformers do not rely on fixed-size receptive fields, enabling them to capture long-range dependencies throughout the entire input image. Critical components of transformer-based detection algorithms encompass the transformer backbone, which is responsible for extracting high-level features from the input image while capturing spatial relationships and contextual information across the image, the object detection head, and the training phase, during which the loss function typically integrates terms for object classification and bounding box regression. The two transformer-based detectors used in this study are as follows:
  (a) *DETR*: DETR (DEtection TRansformer) [26] represents a groundbreaking object detection framework that leverages transformer-based architectures to conduct end-to-end object detection without the need for traditional anchor-based region proposal networks (RPNs) or non-maximum suppression (NMS). The fundamental innovation of DETR resides in its direct set prediction mechanism, wherein it concurrently forecasts object queries alongside their associated class labels and bounding boxes utilising transformer-based encoder-decoder architecture. This transformative approach enables DETR to tackle challenges inherent in conventional object detection methods, such as fixed anchor boxes, arbitrary object counts, and imbalanced class distributions. By leveraging transformers’ ability to capture global context and relationships between objects, DETR achieves competitive performance while offering a unified and flexible framework for object detection tasks.
  (b) *Deformable DETR*: Deformable DETR [27] is an expansion of the original DETR framework. It elevates object detection performance by integrating deformable attention mechanisms into the transformer-based architecture. This innovative approach empowers the model to dynamically adjust the spatial sampling locations of features, facilitating more adaptable and versatile feature extraction. Deformable DETR effectively captures object deformations, scale variations, and occlusions by integrating deformable attention, resulting in enhanced localisation accuracy and resilience in intricate scenes. Additionally, Deformable DETR preserves the end-to-end nature of the DETR framework, enabling seamless integration into existing detection pipelines while achieving state-of-the-art performance in various object detection benchmarks.

### Model training

The model training and implementation were performed using an NVIDIA GeForce RTX 2080 Ti and NVIDIA Tesla V100 GPU. All the models are pre-trained on the COCO dataset [21] and have been fine-tuned on our dataset.

### Evaluation metrics

The evaluation metrics utilised for model evaluation are the typical COCO object detection metrics, such as precision, recall, confusion matrix, mAP50, mAP75, and mAP50-95 [59]. *Precision* measures the ratio of true positives to all positive predictions, indicating the model’s capacity to minimise false positives. Conversely, *recall* assesses the ratio of true positives to all actual positives, indicating the model’s effectiveness in detecting all instances of a class. *Confusion matrix* provides a comprehensive perspective on the results, displaying the numbers of true positives, true negatives, false positives, and false negatives for each class. *mAP50* depicts the mean Average Precision at an *IoU* (Intersection over Union) threshold of 0.5, *mAP75* at an IoU threshold of 0.75, and *mAP50-95* over IoU thresholds of 0.5–0.95 in steps of 0.05.

### Dataset

**Fig 2:**
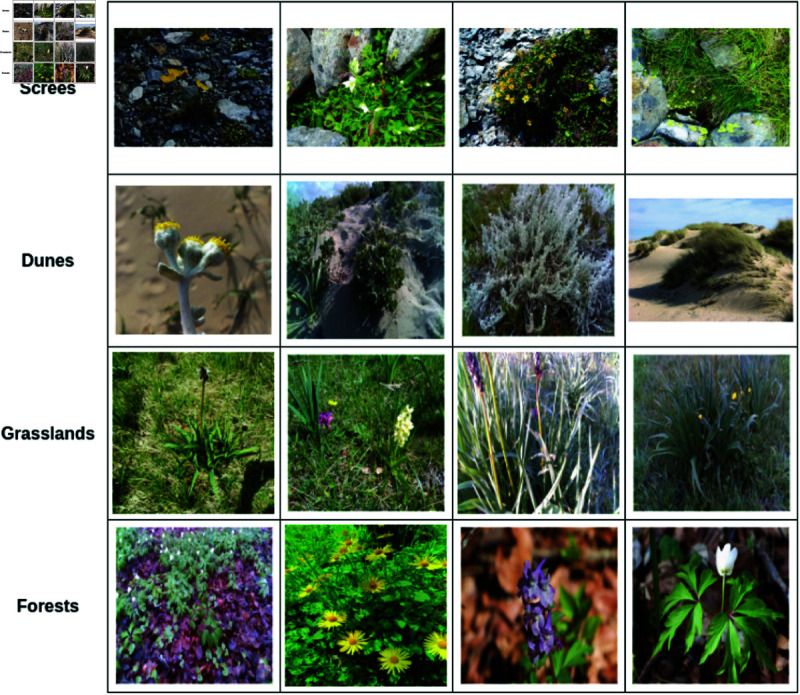
Sample images from each of the four habitats. Only a few of the species considered are displayed in this figure for brevity.

**Fig 3:**
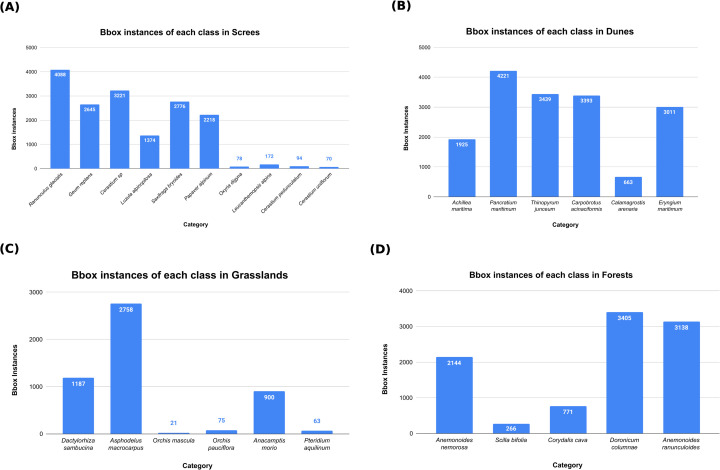
Total bbox instances (class-wise) in training dataset of all habitats.

The dataset includes images of selected target species across four habitats: screes, dunes, grasslands, and forests. Details regarding the data collection process for each habitat: grasslands, screes, forests, and dunes - are provided in [19,60–62], respectively. Expert botanists annotated the collected images by delineating bounding boxes around the relevant plant species using the *Labelbox* tool [20]. Fig 2 presents sample images showcasing four species from each habitat. The dataset was precisely compiled by domain experts who visited the habitat locations and conducted field operations during the appropriate time of the year to capture plant blooming periods. The dataset used in the current study is the combined dataset collected by human operators and the robot. Table 2 lists target plant species from each habitat considered for experimentation. Additionally, Table 3 outlines the distribution of training, validation, and testing data instances, with a split of 70% for training, 20% for validation, and 10% for testing. Fig 3 shows the bounding boxes count of each category in the training dataset of screes, dunes and grasslands, forests, respectively. A class imbalance in all the habitats can be visualised in all the sub-figures. The dataset is available at https://zenodo.org/records/11504938.

**Table 2:** List of target plant species from each habitat used in the study.

Sr.	Screes	Dunes	Grasslands	Forests
1	*Ranunculus glacialis*	*Achillea maritima*	*Dactylorhiza sambucina*	*Anemonoides nemorosa*
2	*Geum reptans*	*Pancratium maritimum*	*Asphodelus macrocarpus*	*Scilla bifolia*
3	*Cerastium* sp	*Thinopyrum junceum*	*Orchis mascula*	*Corydalis cava*
4	*Luzula alpinopilosa*	*Carpobrotus acinaciformis*	*Orchis pauciflora*	*Doronicum columnae*
5	*Saxifraga bryoides*	*Calamagrostis arenaria*	*Anacamptis morio*	*Anemonoides ranunculoides*
6	*Papaver alpinum*	*Eryngium maritimum*	*Pteridium aquilinum*	
7	*Oxyria digyna*			
8	*Leucanthemopsis alpina*			
9	*Cerastium pedunculatum*			
10	*Cerastium uniflorum*			

**Table 3:** Train, validation, and test data splits (images) for each habitat considered for experimentation.

Habitat/Split	Train	Val	Test	Total
Screes	3439	983	492	4914
Dunes	2229	638	319	3186
Grasslands	807	231	116	1154
Forests	1340	384	192	1916
All habitats	7815	2236	1119	11170

### Experimental setup

The specific configuration for each of the models implemented in MMDetection is presented in Table 4. The columns in the table include technique, backbone used in that technique, type of network or attention heads(AHs) and transformer layers (TLs) in case of transformer-based methods, training schedule or duration, learning rate, optimizer, weight decay, momentum, and the maximum number of epochs for which the model has been trained. The model configuration is the same for Faster RCNN, Cascade RCNN, and RetinaNet. The training schedule 1*x* means that the model has been trained for one epoch over the dataset. However, training durations 150e and 50e depict that the model is trained for 150 epochs and 50 epochs over the dataset, in order. Under the *Network* column, *FPN* represents Feature Pyramid Network, AHs and TLs mean Attention Heads and Transformer Layers, respectively. AdamW optimizer has been utilised in case of DETR and Deformable DETR, which is a variant of Adam optimizer. AdamW integrates weight decay directly into its update step. All the models are pre-trained on the COCO dataset.

**Table 4:** Configuration details of the object detection models implemented in MMDetection. Here, FPN = Feature Pyramid Network, AHs = Attention Heads, TLs = Transformer Layers, SGD = Stochastic Gradient Descent. The weight decay value is 0.0001 for all the methods.

Technique	Backbone	Network	Training schedule/ duration	Learning rate	Optimizer	Momentum	Max epochs
Faster RCNN	ResNet-101	FPN	1x	0.02	SGD	0.9	12
Cascade RCNN	ResNet-101	FPN	1x	0.02	SGD	0.9	12
RetinaNet	ResNet-101	FPN	1x	0.02	SGD	0.9	12
DETR	ResNet-50	8AHs, 2TLs	150e	0.0001	AdamW	-	150
Deformable DETR	ResNet-50	16AHs, 2TLs	50e	0.0002	AdamW	-	50

Table 5 shows the YOLOv8 hyperparameter values for diverse model training for all the habitats. The first column has all the hyperparameters, and the second column shows the default values used in YOLOv8 architecture, which are the same for all habitats. The subsequent columns present the different hyperparameter values for two settings: 200*e*, 10*iter* and 100*e*, 20*iter*. The setting 200*e* and 10*iter* means that the hyperparameter tuning has been performed on YOLO for 10 iterations and 200 epochs; similarly, 100*e* and 20*iter* represent 20 iterations and 100 epochs. After getting the best hyperparameters for a particular setting, YOLO was trained separately for each habitat using those hyperparameter values for 200 epochs. YOLO has been trained with default parameters for 500 epochs with the patience of 50 (the number of epochs to wait without any improvement in validation metrics before stopping the training early).

**Table 5:** YOLOv8 hyperparameter values for each setting for all habitats.

Hyper-parameters	Default values	Screes		Dunes		Grasslands		Forests	
		200e, 10iter	100e, 20iter	200e, 10iter	100e, 20iter	200e, 10iter	100e, 20iter	200e, 10iter	100e, 20iter
lr0	0.01	0.00751	0.00471	0.01015	0.01	0.01	0.00803	0.01122	0.00996
lrf	0.01	0.01065	0.00692	0.01219	0.01	0.00789	0.00888	0.00941	0.01027
momentum	0.937	0.98	0.94336	0.96179	0.93252	0.9285	0.937	0.87868	0.93768
weight_decay	0.0005	0.00035	0.00061	0.00043	0.00048	0.00049	0.0005	0.00051	0.00052
warmup_epochs	3	2.51402	4.59436	3.02329	2.98445	3.27	3	3.41974	3.02184
warmup_momentum	0.8	0.67023	0.95	0.95	0.83068	0.88375	0.8	0.79364	0.812
box	7.5	6.28863	6.20141	11.28125	7.10701	7.04156	8.00745	7.79037	7.47292
cls	0.5	0.47166	0.64622	0.44482	0.47202	0.5424	0.5	0.57276	0.50222
dfl	1.5	1.83667	0.88023	1.77283	1.5	1.18402	1.39002	1.04811	1.51298
hsv_h	0.015	0.01165	0.01277	0.01328	0.01482	0.01924	0.01372	0.01379	0.01495
hsv_s	0.7	0.73689	0.57806	0.76946	0.72753	0.61251	0.58956	0.55865	0.69339
hsv_v	0.4	0.34782	0.5041	0.51491	0.40876	0.37126	0.40714	0.51022	0.40214
degrees	0	0	0	0	0	0	0	0	0
translate	0.1	0.11458	0.09543	0.07335	0.09755	0.10086	0.09785	0.10542	0.09868
scale	0.5	0.49414	0.56409	0.66544	0.5	0.58957	0.54626	0.48043	0.50134
shear	0	0	0	0	0	0	0	0	0
perspective	0	0	0	0	0	0	0	0	0
flipud	0	0	0	0	0	0	0	0	0
fliplr	0.5	0.52159	0.48638	0.48671	0.54604	0.52312	0.5	0.4708	0.49588
mosaic	1	0.75364	0.96775	0.67445	1	0.91071	1	1	0.96711
mixup	0	0	0	0	0	0	0	0	0
copy_paste	0	0	0	0	0	0	0	0	0

## Results and discussion

The results presented in this section are all obtained after testing the trained models on test data, and the values in bold depict the highest values in the respective row. The mAP score values based on three different IoU thresholds for screes, dunes, grasslands, and forests habitats are presented in Table 6. The performance of YOLOv8 is the best for all the habitats except dunes, in which RetinaNet has a slightly higher value of mAP and mAP50, and cascade RCNN surpasses in the case of mAP75. *YOLOv8n* depicts the *n* model of YOLOv8 (which is the smallest in size). Cascade RCNN has shown the second-best performance. The reason behind the superior results of YOLOv8 and RetinaNet could be the use of *focal loss* as a loss function, which handles the class imbalance [23]. Cascade RCNN is also performing well due to its multi-stage architecture that refines the proposals in the subsequent stages [25]. Perhaps the transformer-based models (DETR and Deformable DETR) are not performing well due to the requirement of significant training data [27]. DETR has the worst performance as it also requires a considerable number of training epochs to converge [26].

**Table 6:** mAP score results across all habitats (Screes, Dunes, Grasslands, Forests).

Habitat	Metric	Faster RCNN	Cascade RCNN	RetinaNet	YOLOv8n	DETR	Deformable DETR
Screes	mAP	0.2047	0.2195	0.1981	0.228	0.0962	0.2111
	mAP50	0.4007	0.4048	0.3695	0.42	0.1845	0.3912
	mAP75	0.1825	0.1949	0.1855	0.215	0.0833	0.1852
Dunes	mAP	0.276	0.285	0.2888	0.284	0.1635	0.2646
	mAP50	0.5386	0.5205	0.5387	0.521	0.3052	0.4742
	mAP75	0.2372	0.2682	0.2586	0.255	0.1454	0.255
Grasslands	mAP	0.2905	0.3102	0.2744	0.316	0.1027	0.2585
	mAP50	0.6406	0.6553	0.6337	0.667	0.2577	0.6142
	mAP75	0.1812	0.2082	0.1854	0.226	0.0633	0.1711
Forests	mAP	0.42	0.4469	0.42	0.513	0.1429	0.3741
	mAP50	0.7313	0.7417	0.729	0.797	0.3454	0.7073
	mAP75	0.4128	0.4473	0.3992	0.52	0.1085	0.3397

Table 7 shows the mAP scores after incorporating class balancing using *ClassBalancedDataset* wrapper in MMDetection on all habitats. The oversample threshold value (*float* datatype) chosen was 0.1 for all the habitats, considering the instance (bounding box) count of each species in the training dataset of each habitat. This value provides a frequency threshold to the model below which data is repeated. Class balancing has not been performed in YOLOv8 as it is specific to MMDetection, and YOLO also has built-in class balancing to some extent. However, the same YOLOv8 results have been added in Table 7 to show the comparison with other models’ results. We have not utilised any class balancing techniques separately before the models’ implementation. The results obtained after applying ClassBalanced wrapper are mixed. If we look closely, the mAP score values (after class balancing) have decreased primarily in the case of grasslands and forests, which might be due to overfitting as the number of instances in both these habitats is less compared to screes and dunes. For screes and dunes, the scores have increased mostly except for Cascade RCNN. YOLOv8 results are still better than other models (even without applying class balancing separately), except in the case of the dunes habitat, where RetinaNet is leading, but YOLO is close. Cascade RCNN mAP scores are dropped after class balancing, possibly due to overfitting or complex Cascade RCNN architecture. Cascade RCNN has a sequence of stages that refine the proposals [25]. Class balancing might interfere with the progressive refinement process, particularly if some classes are over-represented in the initial stages, resulting in sub-optimal proposals for subsequent stages.

**Table 7:** mAP scores across all habitats (Screes, Dunes, Grasslands, Forests) with class balancing.

Habitat	Metric	Faster RCNN	Cascade RCNN	RetinaNet	YOLOv8n	DETR	Deformable DETR
Screes	mAP	0.2171	0.1238	0.2238	0.228	0.0943	0.2159
	mAP50	0.4095	0.3514	0.4224	0.42	0.1923	0.4196
	mAP75	0.1989	0.0448	0.2051	0.215	0.0748	0.2055
Dunes	mAP	0.2773	0.178	0.2843	0.284	0.1723	0.2746
	mAP50	0.5371	0.4643	0.5339	0.521	0.3256	0.4873
	mAP75	0.2462	0.0883	0.262	0.255	0.1591	0.261
Grasslands	mAP	0.2921	0.1723	0.2426	0.316	0.1145	0.2671
	mAP50	0.6362	0.5427	0.5298	0.667	0.2939	0.6648
	mAP75	0.1878	0.0572	0.1772	0.226	0.0721	0.1616
Forests	mAP	0.4133	0.284	0.4278	0.513	0.1418	0.412
	mAP50	0.7211	0.6765	0.7466	0.797	0.3424	0.7412
	mAP75	0.4038	0.1648	0.3917	0.52	0.1035	0.3925

As YOLOv8 showed better performance than other models even after class balancing, it was chosen for hyperparameter tuning to improve the performance, if possible. So, YOLOv8 model analysis has been performed separately to compare the before (with default parameters) and after hyperparameter tuning results (Table 8). YOLOv8 was executed with default parameters for 500*e* with patience of 50. So, the model executed for different numbers of epochs for all the habitats, which are mentioned in brackets in the first row and third column of each habitat in Table 8. The third and fourth columns display the evaluation metrics values after hyperparameter tuning using *tune* method in YOLOv8 with two different settings: 200*e*, 10*iter* and 100*e*, 20*iter* (described above), respectively. Results have mostly improved after hyperparameter tuning except for forests, where the default parameters of YOLOv8 are the best for this habitat.

**Table 8:** YOLOv8 performance across habitats before and after hyperparameter tuning.

Habitat		Before, 500e given	After_HP_Tuning, 200e	
		Given epoch	200e, 10iter	100e, 20iter
Screes	Precision	0.572 (154e)	0.52	0.518
	Recall	0.414	0.4	0.404
	mAP	0.228	0.274	0.277
	mAP50	0.42	0.457	0.471
	mAP75	0.215	0.282	0.287
Dunes	Precision	0.605 (162e)	0.619	0.641
	Recall	0.484	0.469	0.461
	mAP	0.284	0.348	0.352
	mAP50	0.521	0.553	0.559
	mAP75	0.255	0.346	0.362
Grasslands	Precision	0.755 (237e)	0.681	0.686
	Recall	0.621	0.658	0.605
	mAP	0.316	0.335	0.326
	mAP50	0.667	0.685	0.669
	mAP75	0.226	0.311	0.298
Forests	Precision	0.807 (366e)	0.794	0.773
	Recall	0.734	0.693	0.723
	mAP	0.513	0.5	0.494
	mAP50	0.797	0.774	0.778
	mAP75	0.52	0.519	0.496

**Fig 4:**
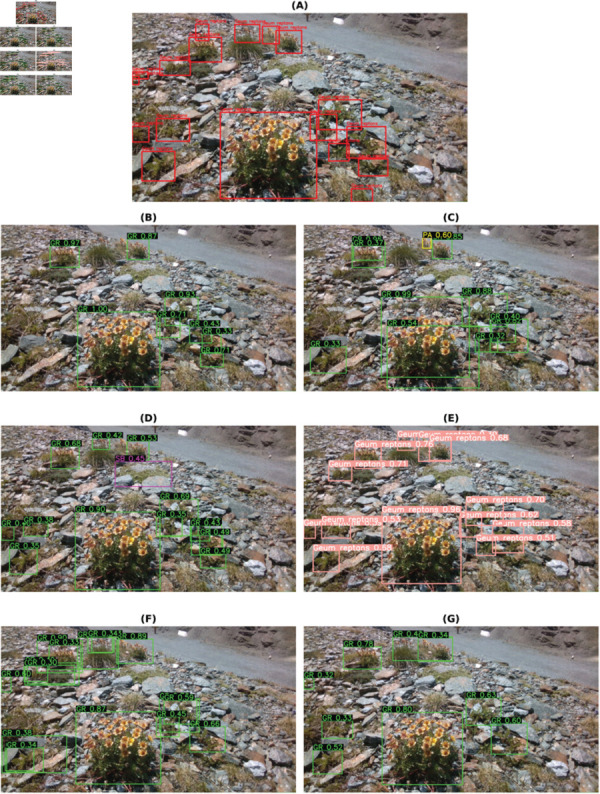
Bounding box predictions from six models on a Screes test image. (A) Ground truth bounding boxes. (B) Faster RCNN. (C) Cascade RCNN. (D) RetinaNet. (E) YOLOv8. (F) DETR. (G) Deformable DETR.

**Fig 5:**
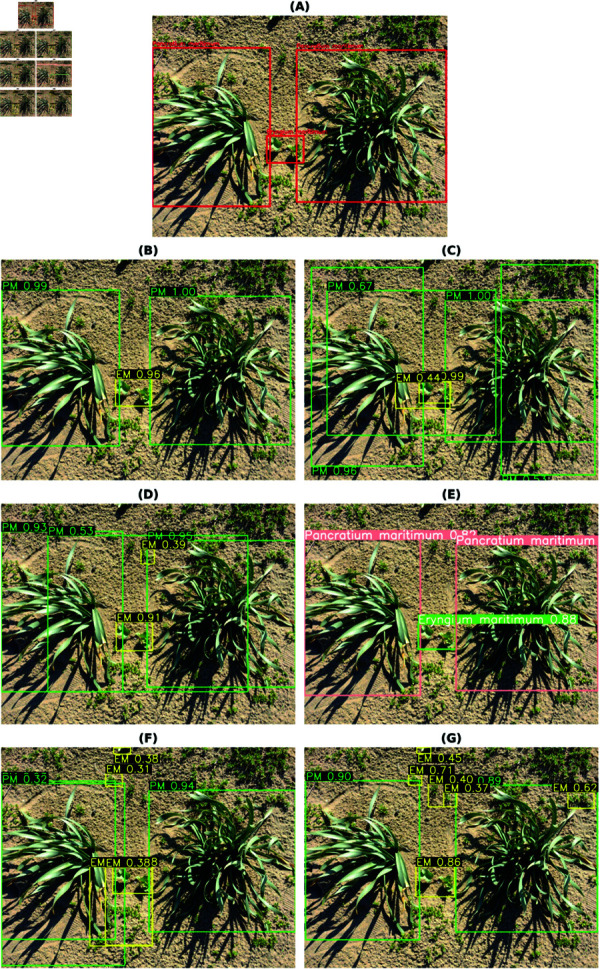
Bounding box predictions from six models on a Dunes test image. (A) Ground truth bounding boxes. (B) Faster RCNN. (C) Cascade RCNN. (D) RetinaNet. (E) YOLOv8. (F) DETR. (G) Deformable DETR.

**Fig 6:**
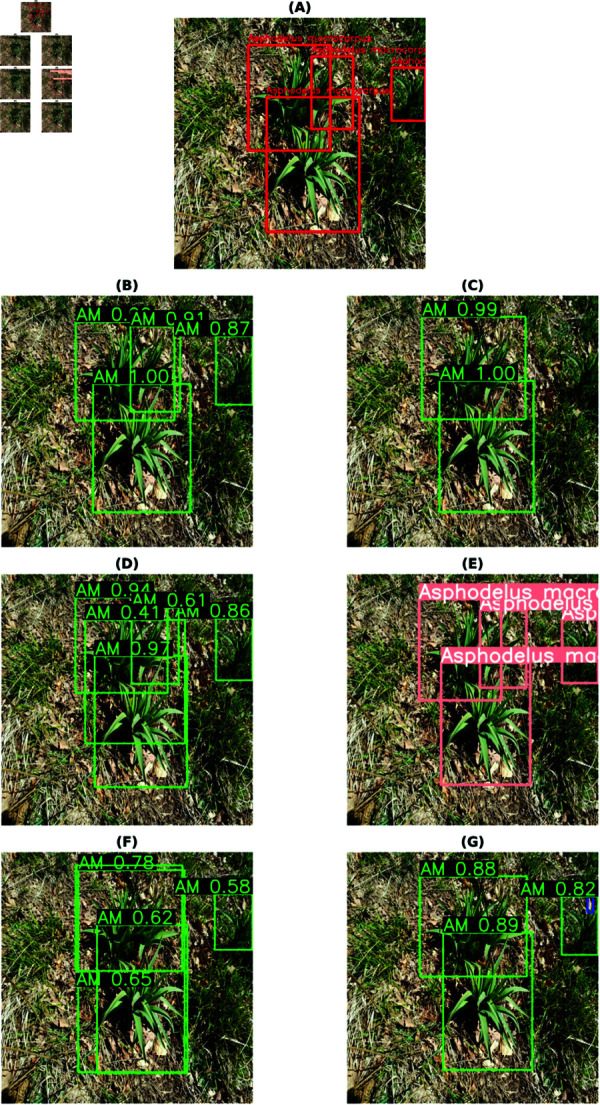
Bounding box predictions from six models on a Grasslands test image. (A) Ground truth bounding boxes. (B) Faster RCNN. (C) Cascade RCNN. (D) RetinaNet. (E) YOLOv8. (F) DETR. (G) Deformable DETR.

**Fig 7:**
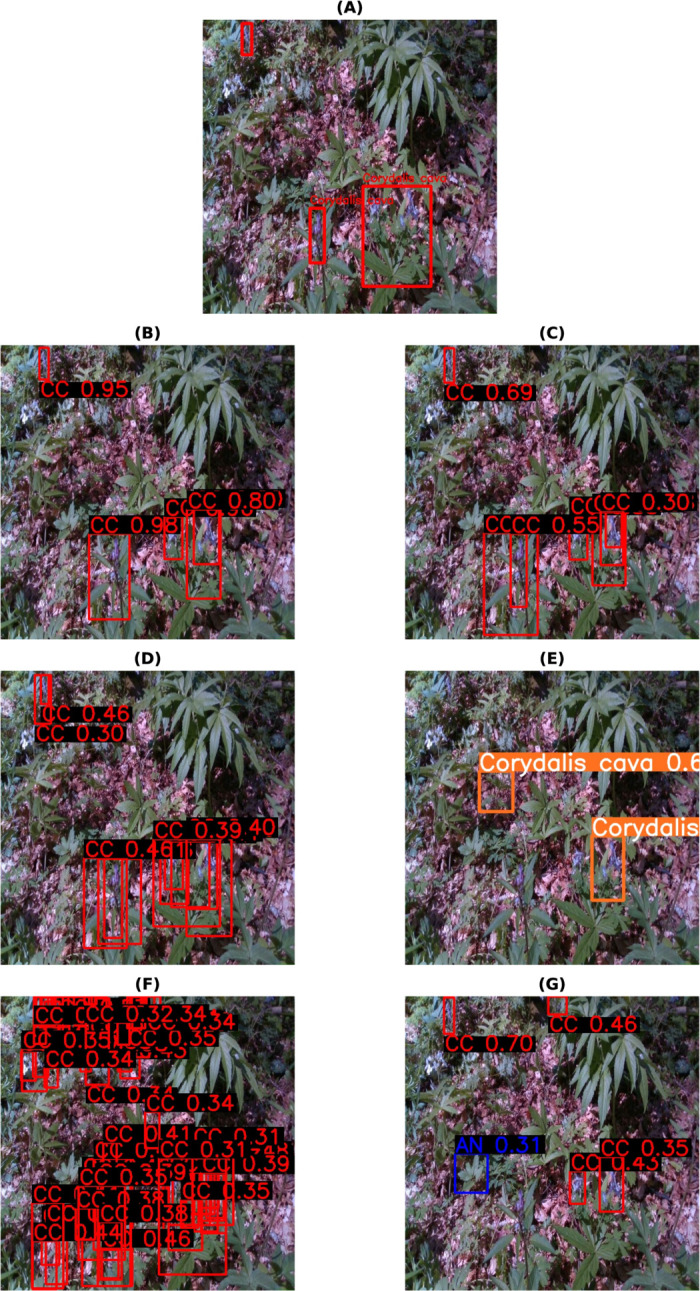
Bounding box predictions from six models on a Forests test image. (A) Ground truth bounding boxes. (B) Faster RCNN. (C) Cascade RCNN. (D) RetinaNet. (E) YOLOv8. (F) DETR. (G) Deformable DETR.

The final best results from all the models for each habitat are shown in Table 9. These are accumulated results from all the above tables, with or without class balancing and hyperparameter tuning. Although the final results are mixed, it can be inferred that most of the best results are with class balancing or hyperparameter tuning (in the case of YOLOv8). YOLO has performed the best out of all the models. So, the class-wise evaluation metrics’ scores have also been shown in the Tables 10–13 obtained from the best models of YOLOv8, respectively, for each habitat. In addition to mAP score, these tables also show the Precision and Recall values. The total number of images (on which the model is tested) for each of the habitats is shown in the *Test* column of Table 3 and the *Instances* column represents the total number of actual bounding boxes of each of the classes in the test images. Figs 4–7 display the predictions of all the models on screes, dunes, grasslands, and forests, along with the ground truth labels (on a single example test image). The classname initials are used with the bounding boxes to show the predictions for better visualisation. The other examples are attached as supporting figures. Below are the inferences deduced from the predicted results:

**Table 9:** Best mAP results across habitats (Screes, Dunes, Grasslands, Forests).

Habitat	Metric	Faster RCNN	Cascade RCNN	RetinaNet	YOLOv8n	DETR	Deformable DETR
Screes		with class_bal	without class_bal	with class_bal	with tune (100e, 20iter)	without class_bal	with class_bal
	mAP	0.2171	0.2195	0.2238	0.2773	0.0962	0.2159
	mAP50	0.4095	0.4048	0.4224	0.4708	0.1845	0.4196
	mAP75	0.1989	0.1949	0.2051	0.2872	0.0833	0.2055
Dunes		with class_bal	without class_bal	without class_bal	with tune (100e, 20iter)	with class_bal	with class_bal
	mAP	0.2773	0.285	0.2888	0.3516	0.1723	0.2746
	mAP50	0.5371	0.5205	0.5387	0.5595	0.3256	0.4873
	mAP75	0.2462	0.2682	0.2586	0.3623	0.1591	0.261
Grasslands		with class_bal	without class_bal	without class_bal	with tune (200e, 10iter)	with class_bal	with class_bal
	mAP	0.2921	0.3102	0.2744	0.3348	0.1145	0.2671
	mAP50	0.6362	0.6553	0.6337	0.6853	0.2939	0.6648
	mAP75	0.1878	0.2082	0.1854	0.3107	0.0721	0.1616
Forests		without class_bal	without class_bal	with class_bal	without tune	without class_bal	with class_bal
	mAP	0.42	0.4469	0.4278	0.5135	0.1429	0.412
	mAP50	0.7313	0.7417	0.7466	0.7975	0.3454	0.7412
	mAP75	0.4128	0.4473	0.3917	0.5204	0.1085	0.3925

**Table 10:** YOLOv8 class-wise results on the Screes habitat.

Class	Instances	Precision	Recall	mAP50	mAP
*Ranunculus glacialis*	452	0.532	0.418	0.486	0.253
*Geum reptans*	380	0.587	0.579	0.587	0.323
*Cerastium sp*	458	0.5	0.332	0.399	0.218
*Luzula alpinopilosa*	216	0.551	0.523	0.583	0.375
*Saxifraga bryoides*	335	0.557	0.496	0.537	0.333
*Papaver alpinum*	258	0.663	0.686	0.724	0.417
*Oxyria digyna*	12	0.8	0.333	0.6	0.253
*Leucanthemopsis alpina*	30	0.429	0.2	0.361	0.232
*Cerastium pedunculatum*	7	0.0588	0.143	0.0541	0.0487
*Cerastium uniflorum*	12	0.5	0.333	0.376	0.321

**Table 11:** YOLOv8 class-wise results on the Dunes habitat.

Class	Instances	Precision	Recall	mAP50	mAP
*Achillea maritima*	316	0.589	0.399	0.477	0.298
*Pancratium maritimum*	661	0.68	0.53	0.612	0.361
*Thinopyrum junceum*	454	0.547	0.207	0.369	0.2
*Carpobrotus acinaciformis*	479	0.627	0.491	0.582	0.367
*Calamagrostis arenaria*	106	0.728	0.557	0.668	0.446
*Eryngium maritimum*	346	0.674	0.581	0.648	0.438

**Table 12:** YOLOv8 class-wise results on the Grasslands habitat.

Class	Instances	Precision	Recall	mAP50	mAP
*Dactylorhiza sambucina*	144	0.737	0.757	0.76	0.367
*Asphodelus macrocarpus*	427	0.757	0.728	0.789	0.491
*Orchis mascula*	6	0.8	0.667	0.766	0.334
*Anacamptis morio*	184	0.744	0.701	0.732	0.295
*Pteridium aquilinum*	16	0.368	0.438	0.38	0.187

**Table 13:** YOLOv8 class-wise results on the Forests habitat.

Class	Instances	Precision	Recall	mAP50	mAP
*Anemonoides nemorosa*	236	0.783	0.843	0.821	0.475
*Scilla bifolia*	40	0.665	0.725	0.738	0.529
*Corydalis cava*	110	0.763	0.555	0.665	0.387
*Doronicum columnae*	571	0.924	0.818	0.899	0.637
*Anemonoides ranunculoides*	345	0.9	0.73	0.864	0.539

1. Almost all models work well for simple predictions (for instance, one or two predictions, plant clearly visible, focus on plant and blurred background).
2. In all four habitats, YOLO has mostly predicted the exact bounding boxes as ground truth labels and with good confidence scores.
3. DETR has provided a large number of detections every time in comparison to the ground truths, for instance, multiple bounding boxes of varied sizes for predicting a single plant species.
4. In the case of screes, the order of models considering well-predicted labels is YOLO, Deformable DETR, Cascade RCNN, Faster RCNN, RetinaNet, and DETR.
5. In the dunes case, the order is YOLO, Cascade RCNN, Faster RCNN, RetinaNet, Deformable DETR, and DETR.
6. In the case of grasslands, the predicted labels by all models are almost the same and also match with the ground truth labels. However, YOLO has shown the best performance, after that, Faster RCNN, RetinaNet and Deformable DETR have similar performances, then comes DETR and Cascade RCNN close predictions. The reason behind these grasslands habitat results could be the simple test examples compared to other habitats.
7. In the case of Forests, the prediction performance of Cascade RCNN is slightly better than YOLO. As most of the plants/bounding boxes are very small, Cascade RCNN is performing very well because of its better small object detection capability due to improvement in localisation by refining bounding box predictions in consecutive Cascade RCNN stages [25]. The performance of Faster RCNN and RetinaNet is close. Then, Deformable DETR and DETR come last.

**Fig 8:**
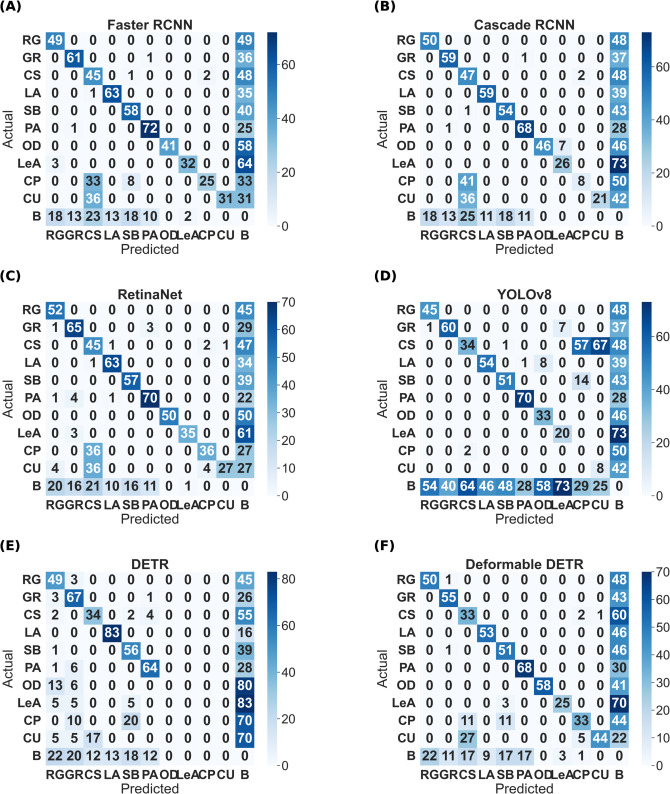
Screes test data confusion matrix of all models. The numbers in the confusion matrices represent percentages.

**Fig 9:**
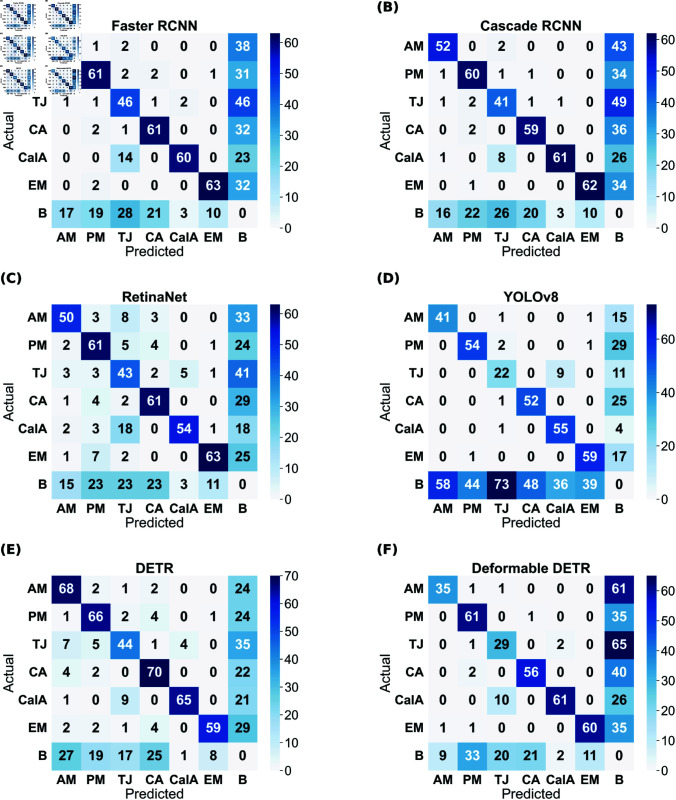
Dunes test data confusion matrix of all models. The numbers in the confusion matrices represent percentages.

**Fig 10:**
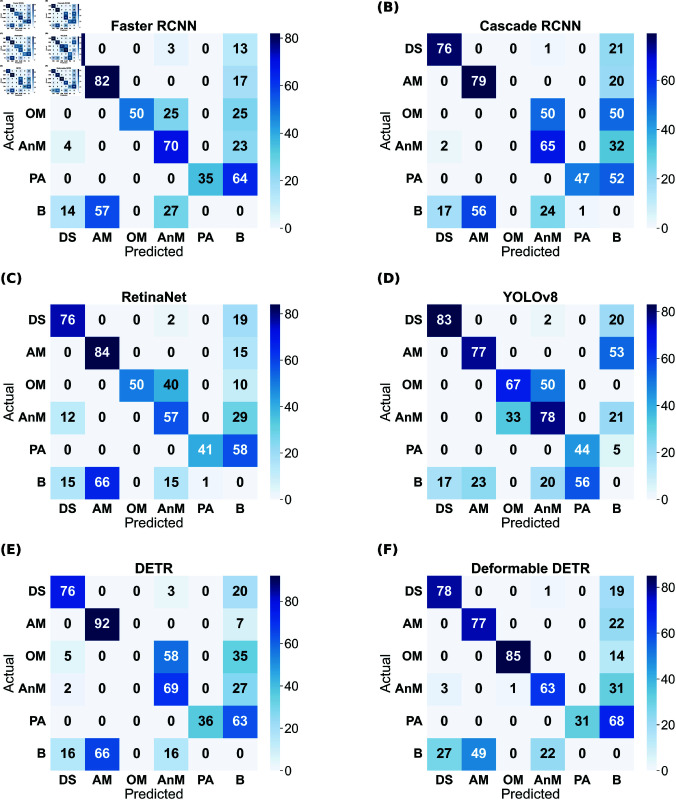
Grasslands test data confusion matrix of all models. The numbers in the confusion matrices represent percentages.

**Fig 11:**
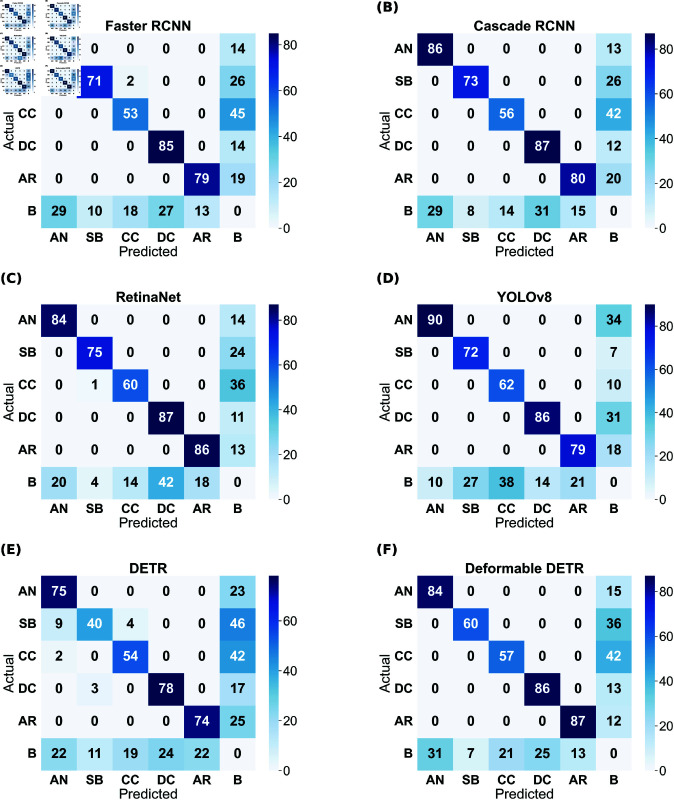
Forests test data confusion matrix of all models. The numbers in the confusion matrices represent percentages.

Although the mAP scores of YOLO are best in all cases, that does not directly imply that other models have inferior performance. It is evident from the prediction results shown in Figs 4– 7 that other models have shown good results. YOLO exactly matches the ground truth in most cases. However, it seems that the two-stage detectors, especially Cascade RCNN, may also predict/find the species correctly, which the botanists might have missed or not annotated in the images during the labelling procedure. Sometimes, it is not possible to annotate each and every instance of plant species in an image due to various reasons, such as a large number of species instances, very small plant size, species in groups, and species not visible properly. Figs 8–11 display the confusion matrices of all the best models (with or without class balancing or hyperparameter tuning) as sub-figures for screes, dunes, grasslands, and forests, respectively. The classname initials have been used in the confusion matrices rather than full names to reduce the space. From all the confusion matrices, it can be inferred that the models are getting more confused between the actual category and the background rather than among different categories. There are two major reasons behind this: (1) similarity among different categories and the background, and (2) sometimes, models have also detected the plant species in the background which were not annotated by the botanists, perhaps due to less clarity and very small plant size. In the case of grasslands in Fig 10, the confusion matrix results for the plant species *Orchis pauciflora* are not included as there is no instance of this class in the test dataset due to a significantly smaller number of instances.

## Conclusion

This article presented an experimental study of the performance of six popular object detection algorithms such as Faster RCNN and Cascade RCNN (two-stage), RetinaNet and YOLOv8 (one-stage), and DETR and Deformable DETR (transformer-based) for plant species identification in four habitats: screes, dunes, grasslands, and forests. After extensive experimentation on all four datasets, the results are discussed. As per the mAP scores, YOLOv8n has been found to be the best-performing model among all, given the ground truth labels. Although YOLOv8 is best in detecting all the categories in an image with nearly exact bounding boxes as per ground truths, other models are also detecting a few of the plant species in the image which were missed during the annotation procedure. As these are considered false positives, so they affect the overall precision, recall, and mAP score values. Confusion matrices and predicted results are also presented in this study for more clarity. There are a few issues in this experimental study: (1) one major issue is the class imbalance, (2) small datasets, (3) limited data diversity, and (4) integration of human-collected and robot-collected data (which was done to increase the instances in the dataset and for incorporating diversity). The data integration might have impacted the results because of the differences in how pictures were taken by human experts and robots. Moreover, for deep learning models to work well, it is advisable to have huge datasets with high and nearly equal number of instances of each class, including diverse examples of each category. Although the class balancing wrapper (in MMDetection) has been used to avoid the class imbalance issue, it did not work very well. In the future, we will attempt to balance the data by using a custom data augmentation technique inspired by state-of-the-art methods such as generative adversarial networks and diffusion models, which can improve plant species identification.

## Supporting information

S1 Fig Bounding box predictions from six models on a Screes test image1. (A) Ground truth bounding boxes. (B) Faster RCNN. (C) Cascade RCNN. (D) RetinaNet. (E) YOLOv8. (F) DETR. (G) Deformable DETR.
(JPG)

S2 Fig Bounding box predictions from six models on a Screes test image2. (A) Ground truth bounding boxes. (B) Faster RCNN. (C) Cascade RCNN. (D) RetinaNet. (E) YOLOv8. (F) DETR. (G) Deformable DETR.
(JPG)

S3 Fig Bounding box predictions from six models on a Dunes test image1. (A) Ground truth bounding boxes. (B) Faster RCNN. (C) Cascade RCNN. (D) RetinaNet. (E) YOLOv8. (F) DETR. (G) Deformable DETR.
(JPG)

S4 Fig Bounding box predictions from six models on a Dunes test image2. (A) Ground truth bounding boxes. (B) Faster RCNN. (C) Cascade RCNN. (D) RetinaNet. (E) YOLOv8. (F) DETR. (G) Deformable DETR.
(JPG)

S5 Fig Bounding box predictions from six models on a Grasslands test image1. (A) Ground truth bounding boxes. (B) Faster RCNN. (C) Cascade RCNN. (D) RetinaNet. (E) YOLOv8. (F) DETR. (G) Deformable DETR.
(JPG)

S6 Fig Bounding box predictions from six models on a Grasslands test image2. (A) Ground truth bounding boxes. (B) Faster RCNN. (C) Cascade RCNN. (D) RetinaNet. (E) YOLOv8. (F) DETR. (G) Deformable DETR.
(JPG)

S7 Fig Bounding box predictions from six models on a Forests test image1. (A) Ground truth bounding boxes. (B) Faster RCNN. (C) Cascade RCNN. (D) RetinaNet. (E) YOLOv8. (F) DETR. (G) Deformable DETR.
(JPG)

S8 Fig Bounding box predictions from six models on a Forests test image2. (A) Ground truth bounding boxes. (B) Faster RCNN. (C) Cascade RCNN. (D) RetinaNet. (E) YOLOv8. (F) DETR. (G) Deformable DETR.
(JPG)
