# Supplementary figures and images for: Artificial vision models for the identification of Mediterranean flora: An analysis in four ecosystems
Source: PLoS One. 2025 Sep 5;20(9):e0327969. doi: 10.1371/journal.pone.0327969 (PMC12413090; doi:10.1371/journal.pone.0327969)

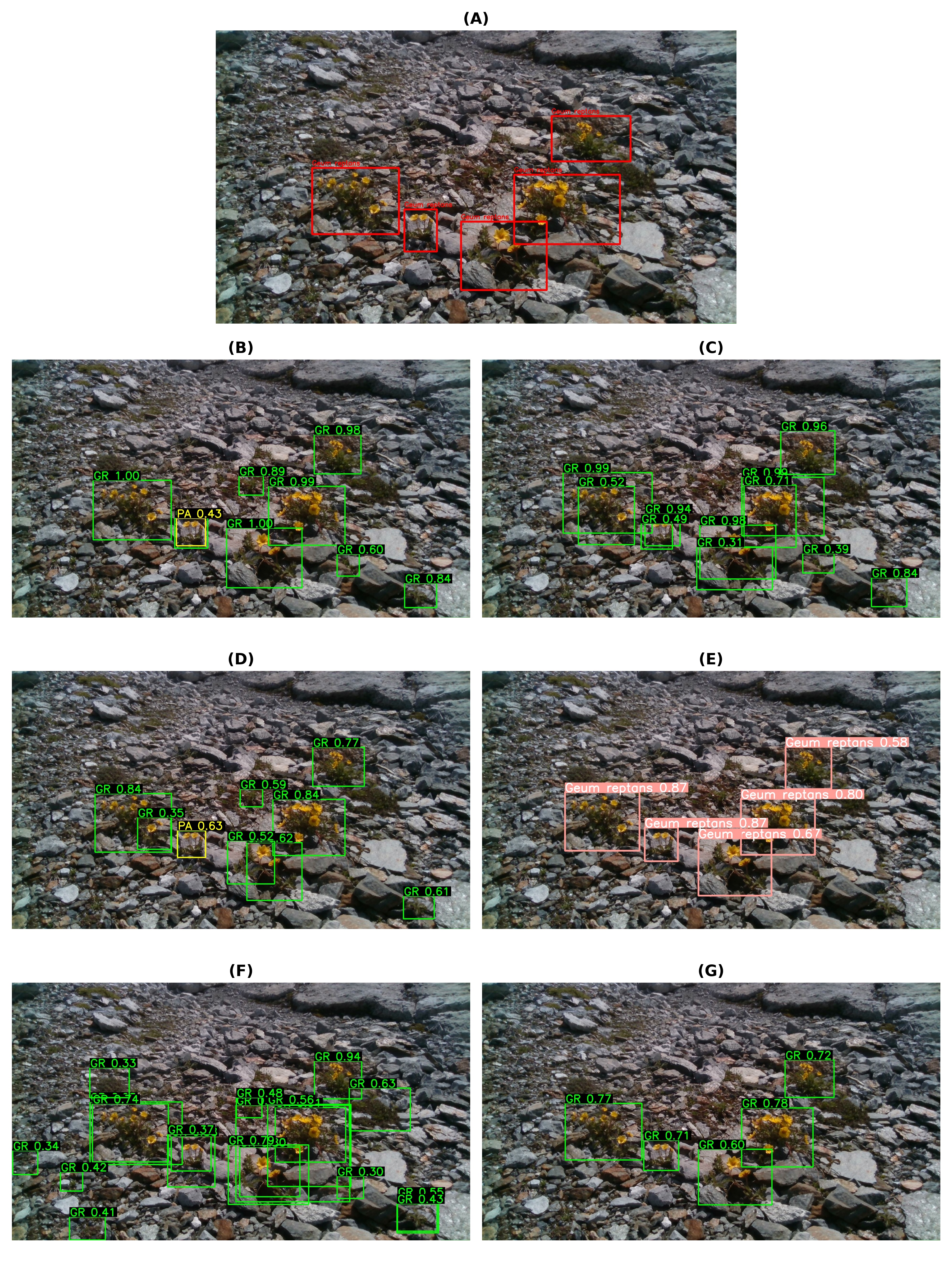

Supplement: S1 Fig — (A) Ground truth bounding boxes. (B) Faster RCNN. (C) Cascade RCNN. (D) RetinaNet. (E) YOLOv8. (F) DETR. (G) Deformable DETR. (JPG) [file pone.0327969.s001.jpg]

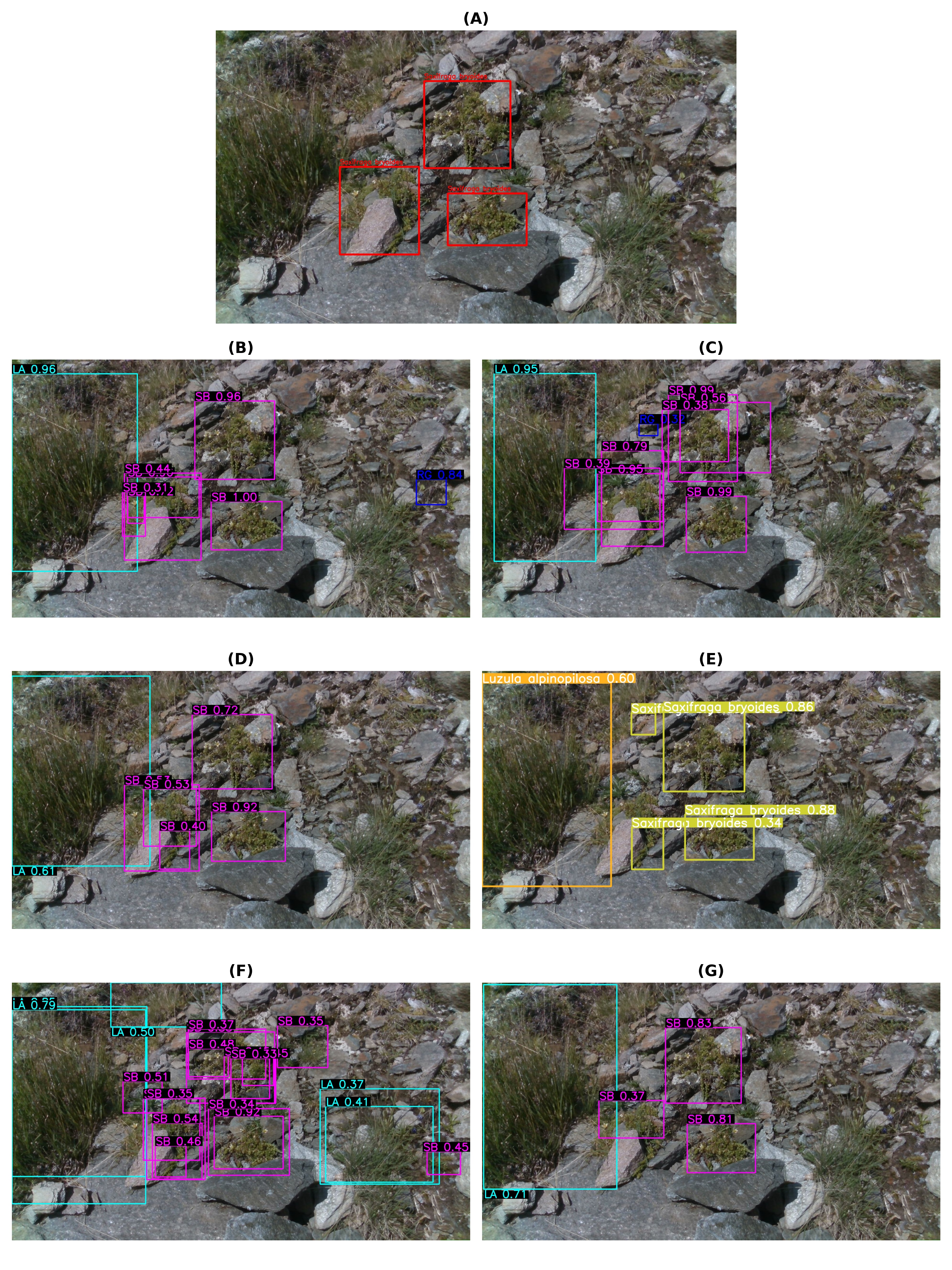

Supplement: S2 Fig — (A) Ground truth bounding boxes. (B) Faster RCNN. (C) Cascade RCNN. (D) RetinaNet. (E) YOLOv8. (F) DETR. (G) Deformable DETR. (JPG) [file pone.0327969.s002.jpg]

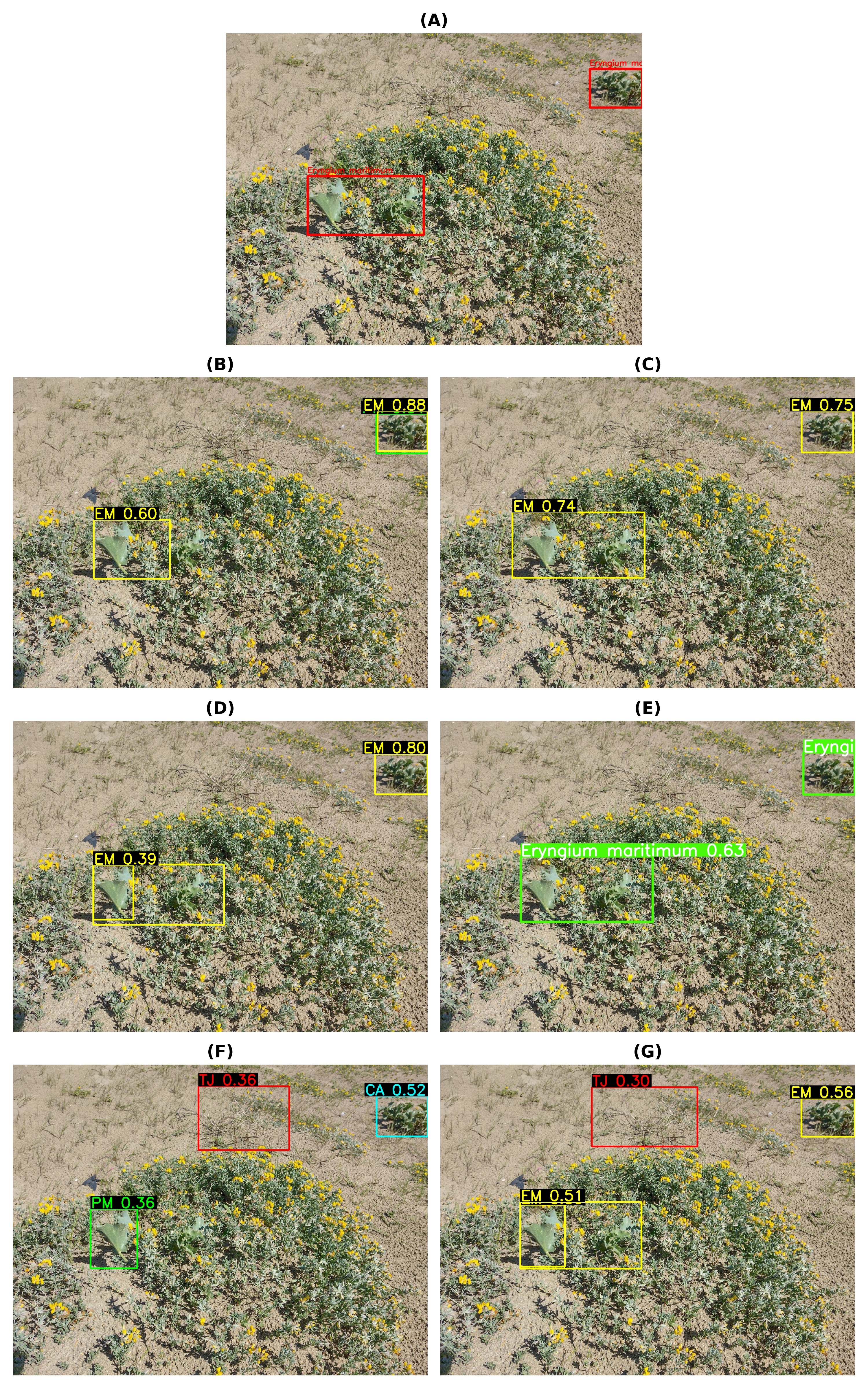

Supplement: S3 Fig — (A) Ground truth bounding boxes. (B) Faster RCNN. (C) Cascade RCNN. (D) RetinaNet. (E) YOLOv8. (F) DETR. (G) Deformable DETR. (JPG) [file pone.0327969.s003.jpg]

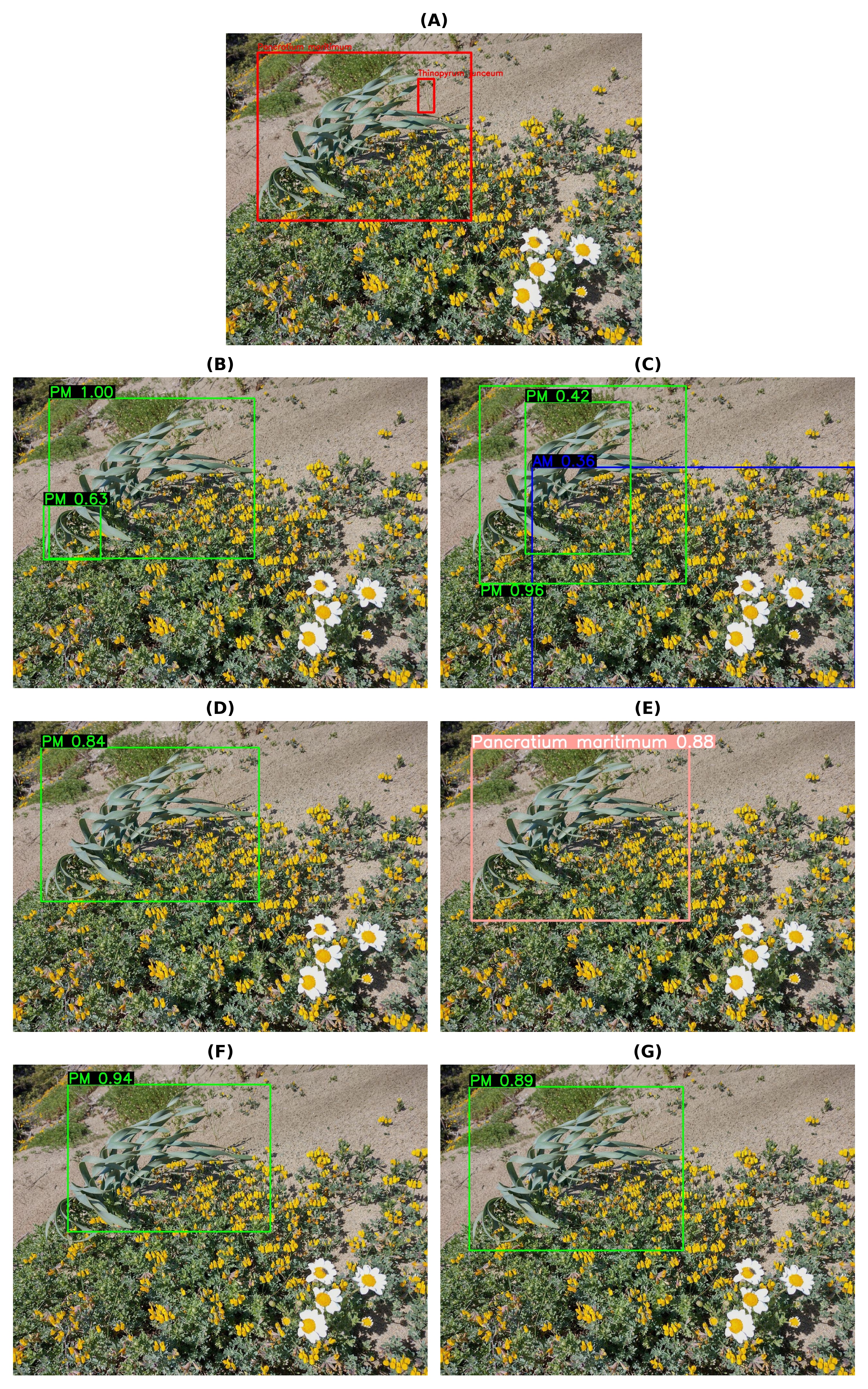

Supplement: S4 Fig — (A) Ground truth bounding boxes. (B) Faster RCNN. (C) Cascade RCNN. (D) RetinaNet. (E) YOLOv8. (F) DETR. (G) Deformable DETR. (JPG) [file pone.0327969.s004.jpg]

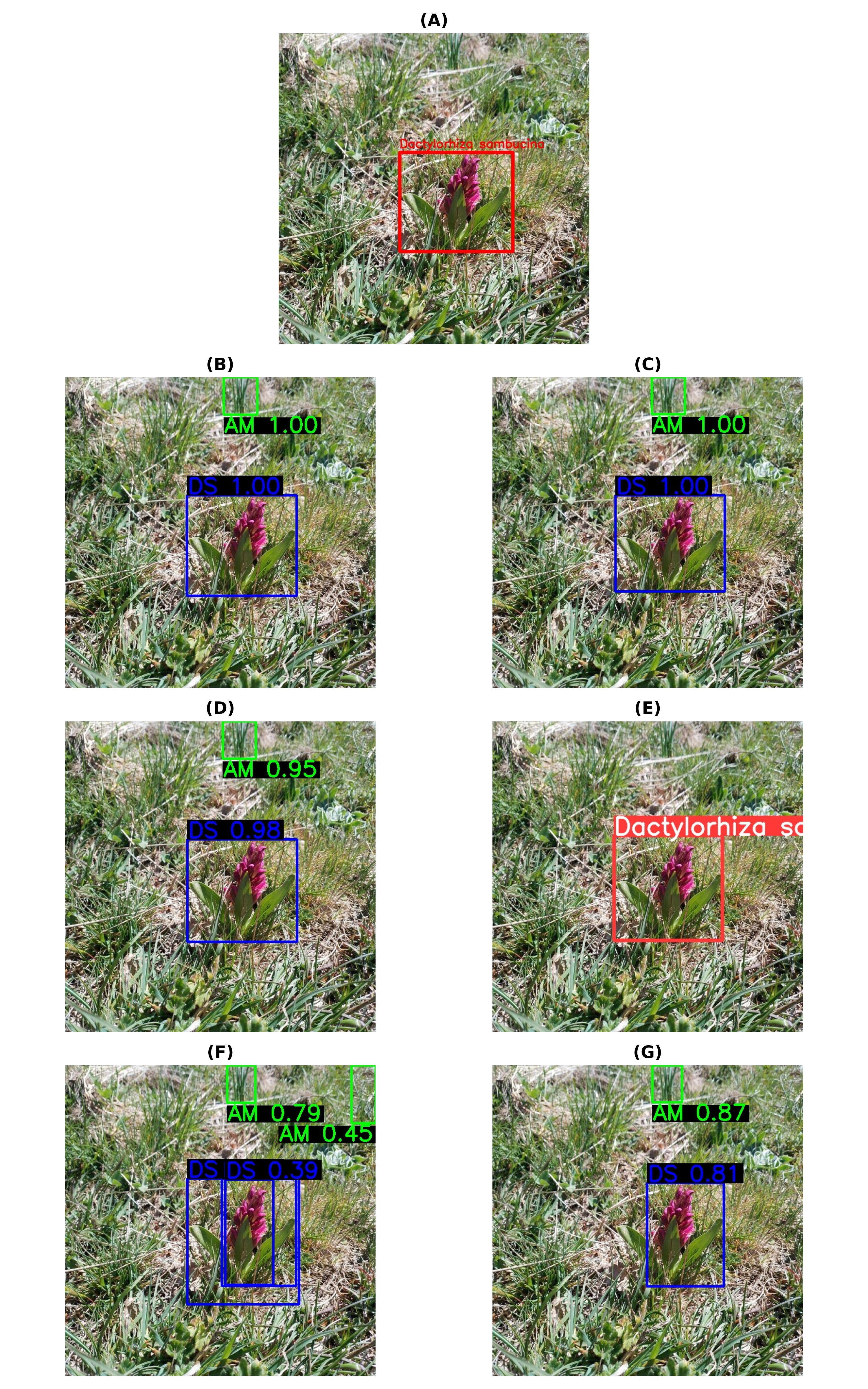

Supplement: S5 Fig — (A) Ground truth bounding boxes. (B) Faster RCNN. (C) Cascade RCNN. (D) RetinaNet. (E) YOLOv8. (F) DETR. (G) Deformable DETR. (JPG) [file pone.0327969.s005.jpg]

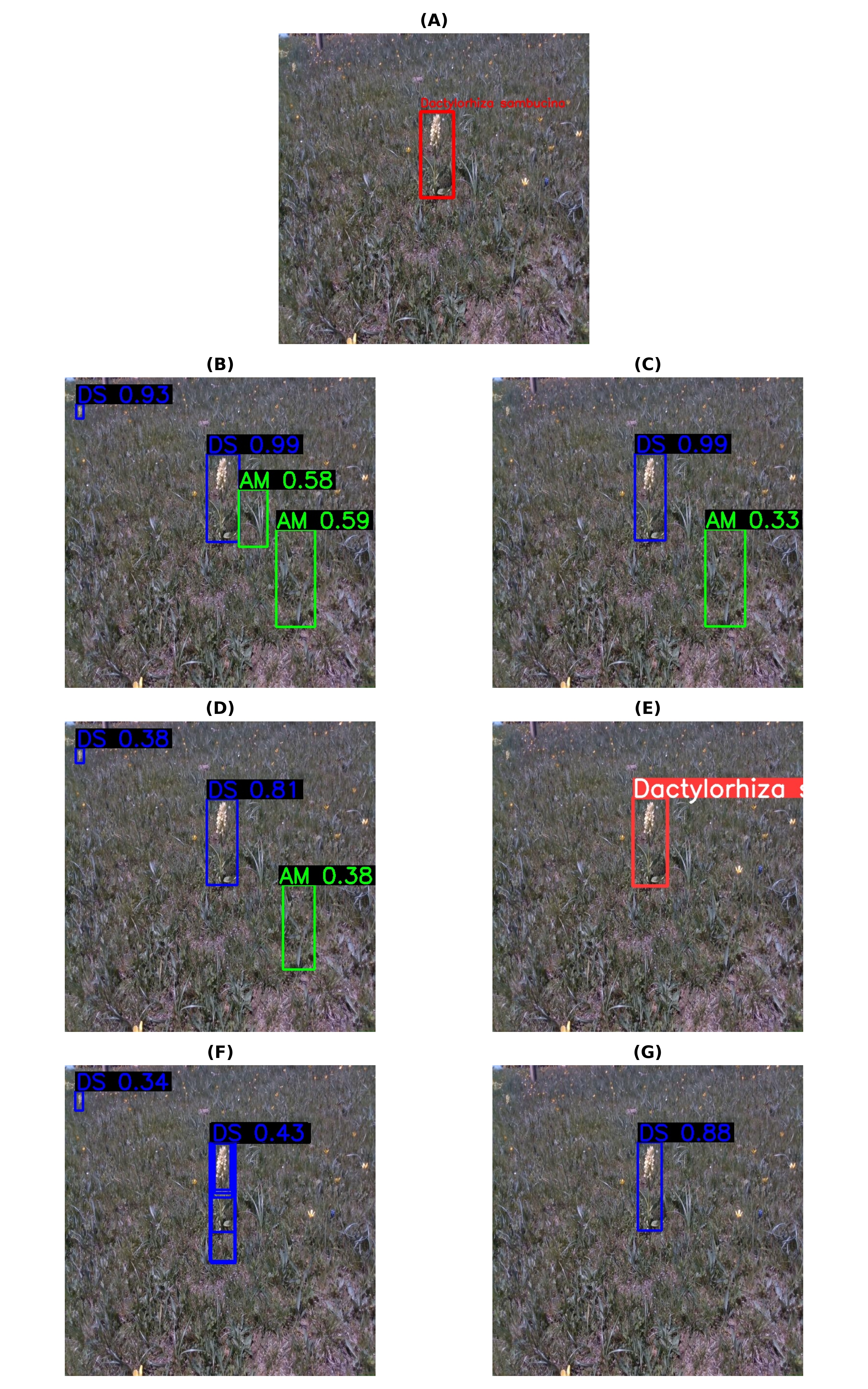

Supplement: S6 Fig — (A) Ground truth bounding boxes. (B) Faster RCNN. (C) Cascade RCNN. (D) RetinaNet. (E) YOLOv8. (F) DETR. (G) Deformable DETR. (JPG) [file pone.0327969.s006.jpg]

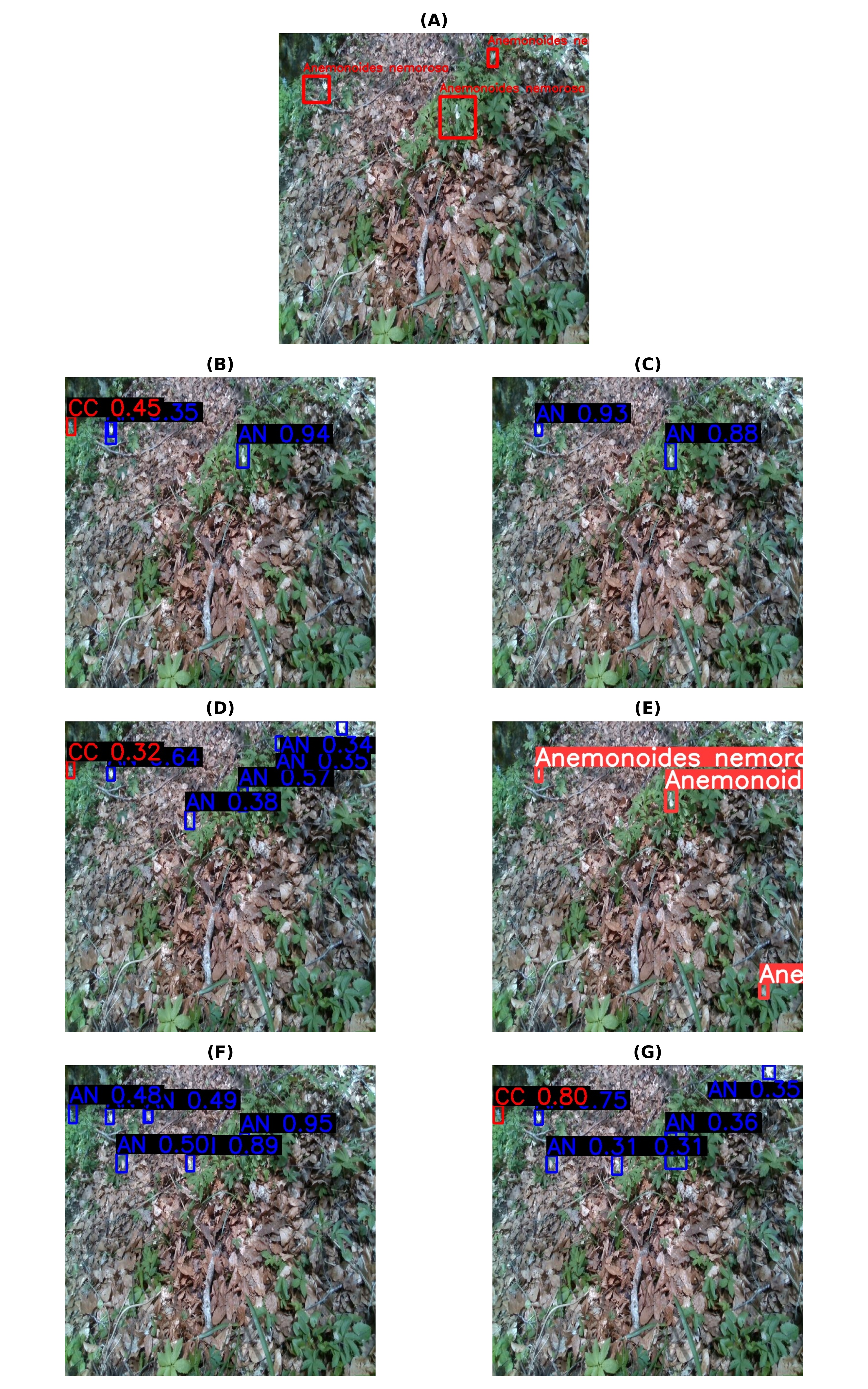

Supplement: S7 Fig — (A) Ground truth bounding boxes. (B) Faster RCNN. (C) Cascade RCNN. (D) RetinaNet. (E) YOLOv8. (F) DETR. (G) Deformable DETR. (JPG) [file pone.0327969.s007.jpg]

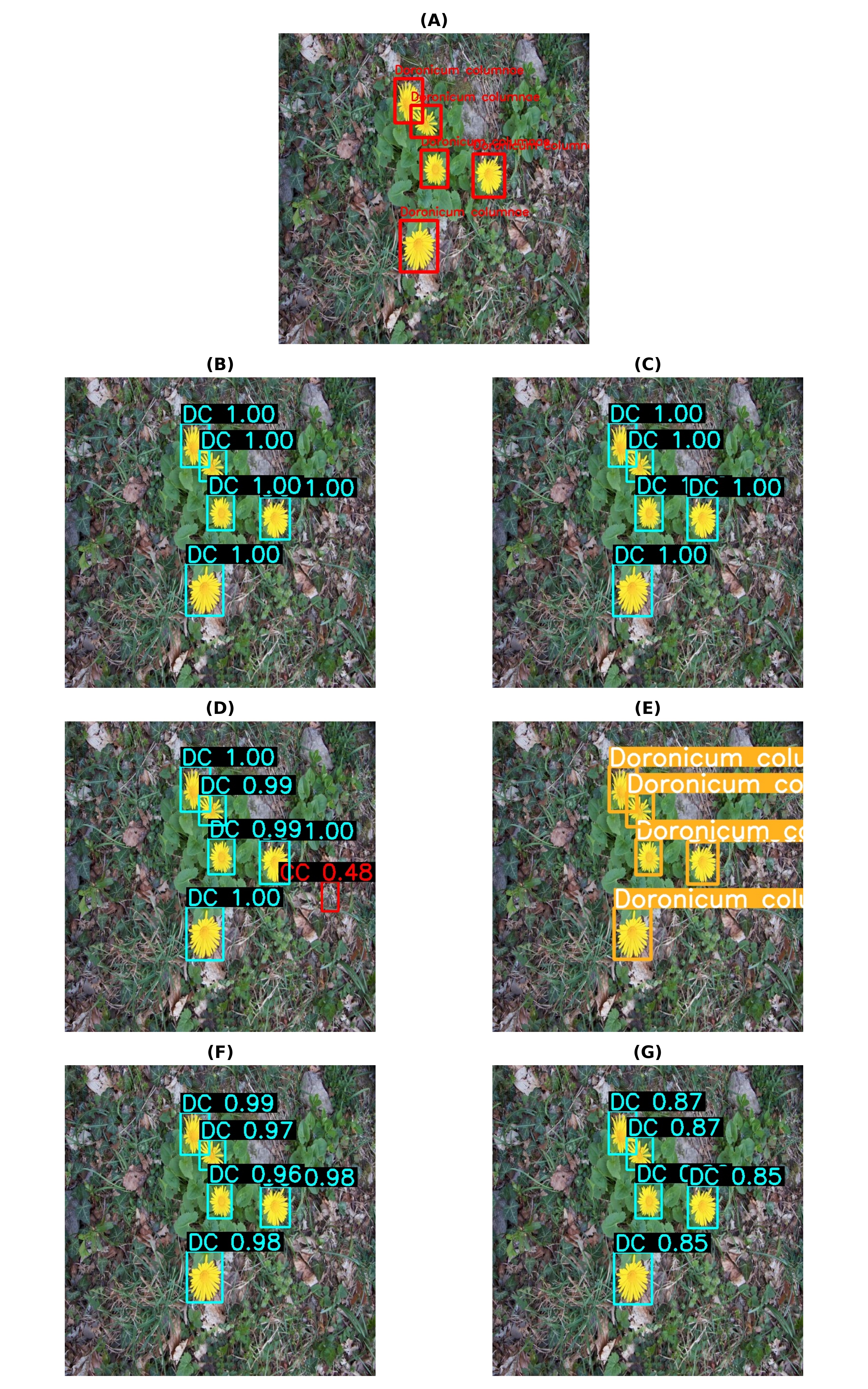

Supplement: S8 Fig — (A) Ground truth bounding boxes. (B) Faster RCNN. (C) Cascade RCNN. (D) RetinaNet. (E) YOLOv8. (F) DETR. (G) Deformable DETR. (JPG) [file pone.0327969.s008.jpg]
